# Supplementary material for: Characterizing the roles of bots on Twitter during the COVID-19 infodemic
Source: J Comput Soc Sci. 2021 Aug 30;5(1):591–609. doi: 10.1007/s42001-021-00139-3 (PMC8403696; doi:10.1007/s42001-021-00139-3)
Supplement: Supplementary file 1 — Supplementary file1 (PDF 320 KB) [file 42001_2021_139_MOESM1_ESM.pdf]

Supplementary Information for characterizing the roles of bots during COVID-19 infodemic on Twitter

Wentao Xu[1] · Kazutoshi Sasahara[2]

1 CCDF

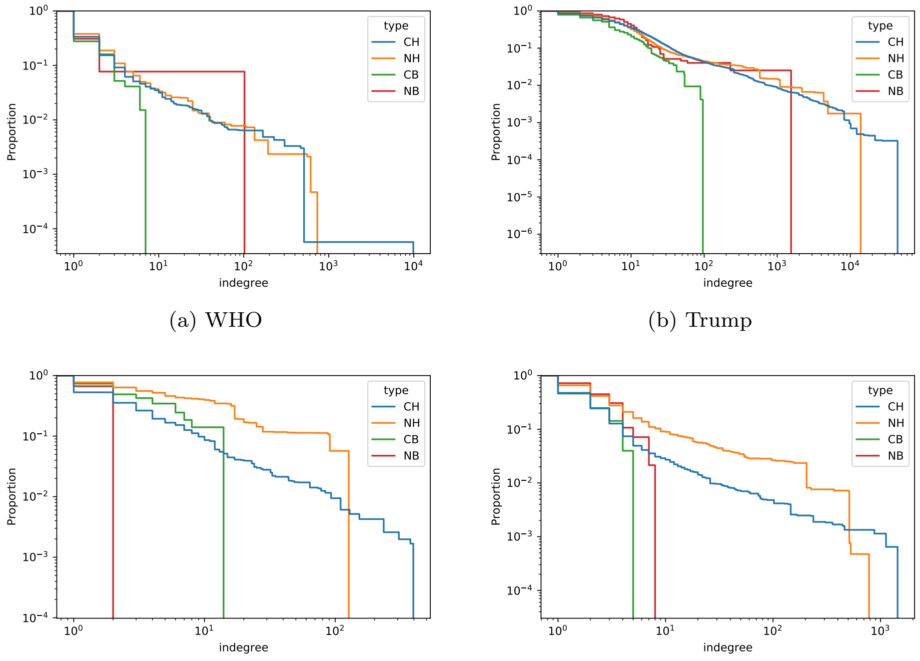

(a) WHO (b) Trump

(c) 5G (d) Bill Gates

Fig. 1: CCDF curves for users of each topic.

E-mail: myrainbowandsky@gmail.com

[1] Graduate School of Informatics, Nagoya University, Japan
[2] School of Environment and Society, Tokyo Institute of Technology, Japan

2 Term importance for each topic

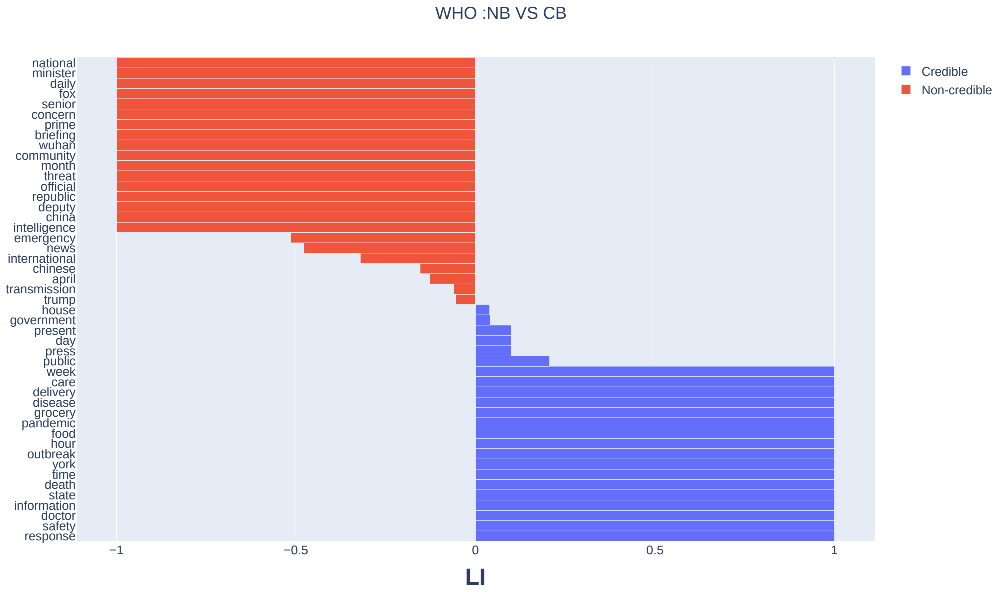

(a)

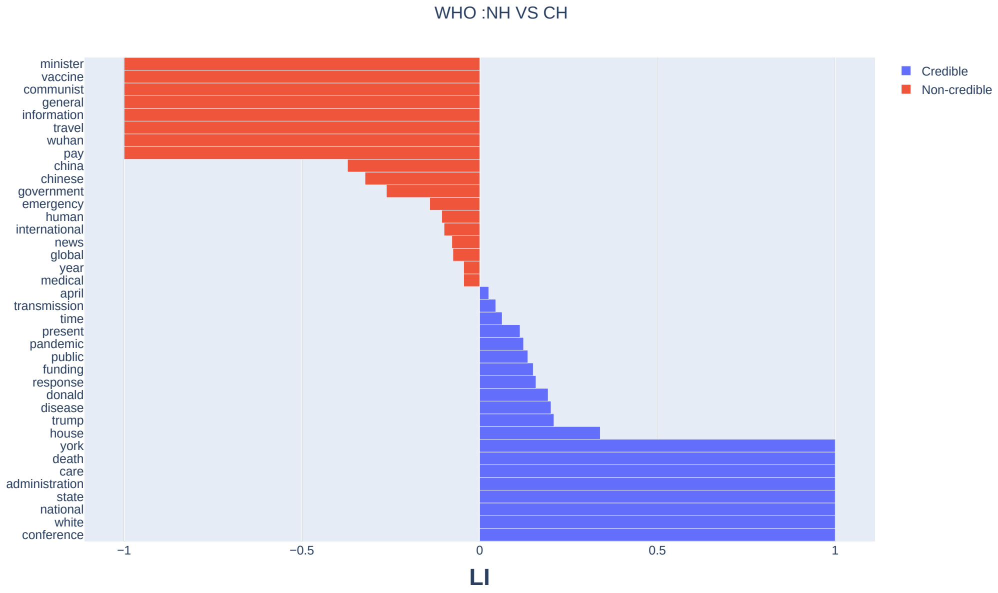

(b)

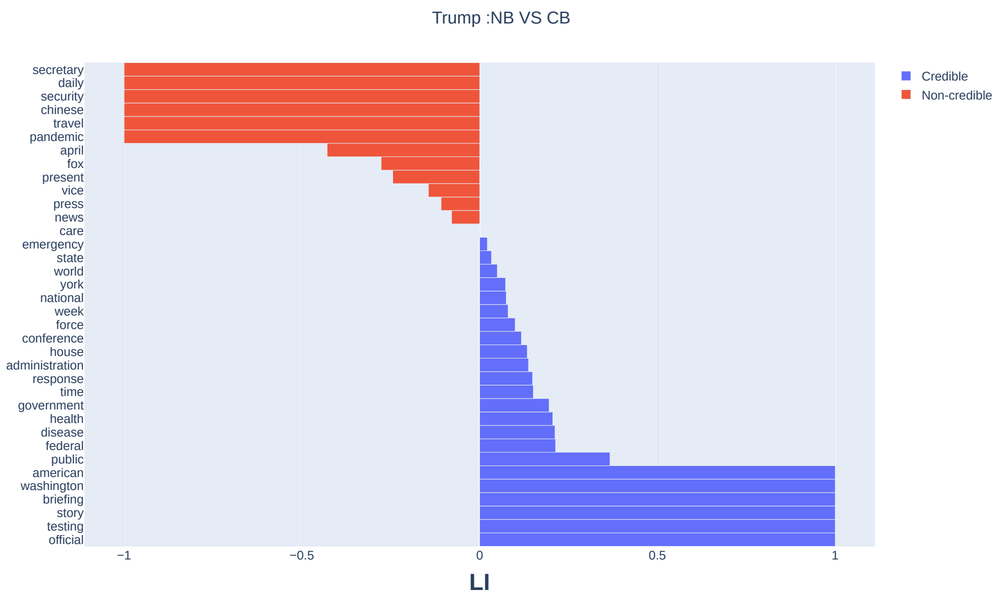
Trump :NB VS CB
secretary
daily
security
chinese
travel
pandemic
april
fox
present
vice
press
news
care
emergency
state
world
york
national
week
force
conference
house
administration
response
time
government
health
disease
federal
public
american
washington
briefing
story
testing
official
−1
−0.5
0
0.5
1
LI
Credible
Non-credible

(c)

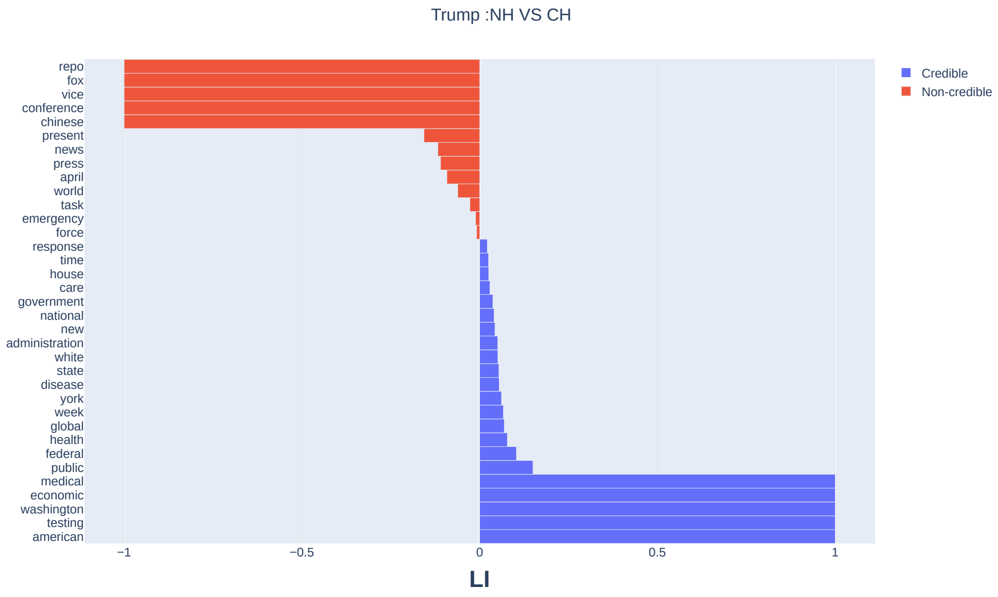
Trump :NH VS CH
repo
fox
vice
conference
chinese
present
news
press
april
world
task
emergency
force
response
time
house
care
government
national
new
administration
white
state
disease
york
week
global
health
federal
public
medical
economic
washington
testing
american
−1
−0.5
0
0.5
1
LI
Credible
Non-credible

(d)

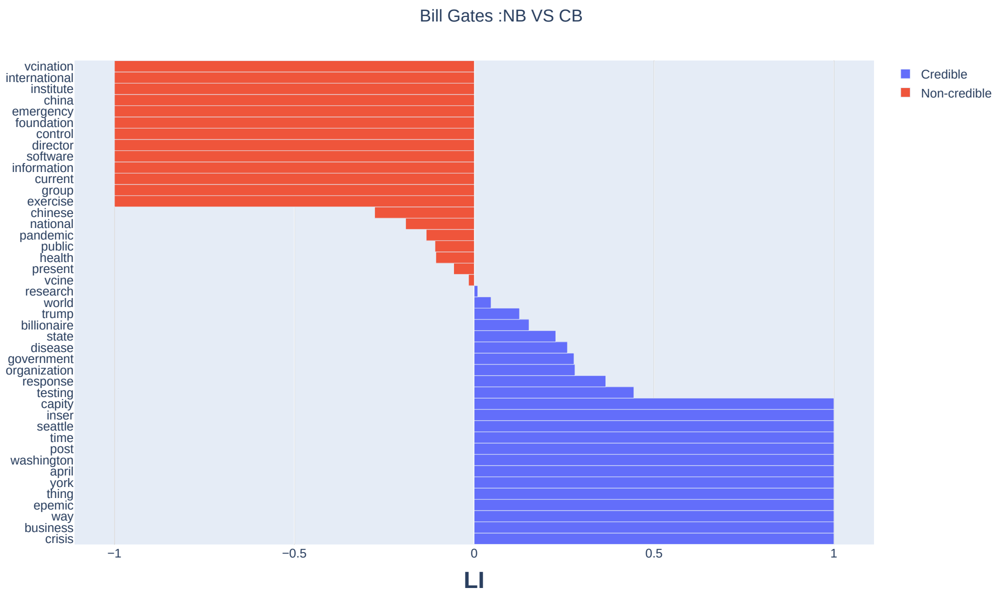

(e)

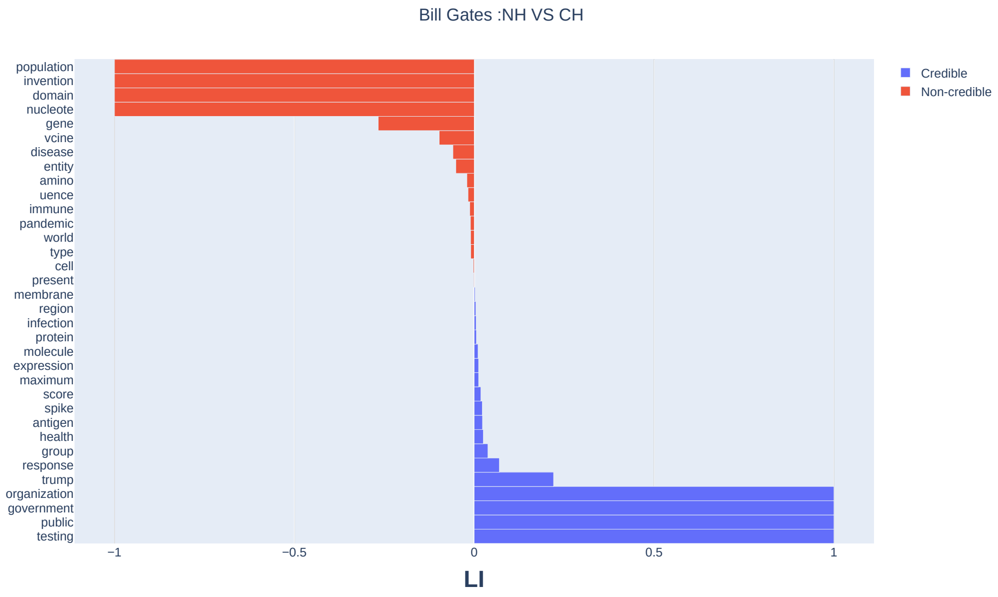

(f)

Fig. 2: Top 30 featured terms by importance ranking of credible users and non-credible users' articles of "WHO", "Trump",and "Bill Gates" topics. Red bars indicate the term was from the articles retweeted by non-credible users; while blue bars indicate the term was from the articles retweeted by credible users. (We selected the top 30 terms from credible users category and top 30 terms from non-credible users category and then merged them without duplicates.)

3 Retweeted domains and users

Top 10 retweeted domains and users favored by credible/non-credible humans and bots. Green for credible users; red for non-credible users; blues for others. Column labels indicate the user type who retweeted.

Table 1: WHO

(a) Top 10 domains retweeted by

	NB	CB	NH	CH
**1**	dailycaller.com	wsj.com	dailycaller.com	wsj.com
**2**	thegatewaypundit.com	reuters.com	thegatewaypundit.com	theguardian.com
**3**	wikileaks.org	theguardian.com	wikileaks.org	cnn.com
**4**	foxnews.com	bbc.com	presstv.com	washingtonpost.com
**5**	ahtribune.com	nytimes.com	ahtribune.com	nytimes.com
**6**	presstv.com	bbc.co.uk	foxnews.com	reuters.com
**7**	rt.com	cnn.com	breitbart.com	bbc.com
**8**	zerohedge.com	usatoday.com	justthenews.com	newsweek.com
**9**	saraacarter.com	washingtonpost.com	politicususa.com	bbc.co.uk
**10**	collective-evolution.com	foxnews.com	lifesitenews.com	justthenews.com

(b) Top 10 users retweeted by

	NB	CB	NH	CH
**1**	@gatewaypundit	@KimStrassel	@gatewaypundit	@KimStrassel
**2**	@ouchinagirl	@AmazonOmy	@wikileaks	@guardian
**3**	@wikileaks	@Vastuullisuus	@DailyCaller	@newtgingrich
**4**	@DailyCaller	@Reuters	@ouchinagirl	@jsolomonReports
**5**	@RoseGeorossi	@chidambara09	@Ian56789	@Newsweek
**6**	@Ian56789	@guardian	@PressTV	@chidambara09
**7**	@anuraag_saxena	@newtgingrich	@1776Stonewall	@TheElders
**8**	@PressTV	@jsolomonReports	@anuraag_saxena	@Reuters
**9**	@Doodisgirl	@ABC	@jsolomonReports	@mitchellvii
**10**	@RT_com	@verge	@Doodisgirl	@RWPUSA

Table 2: Trump

(a) Top 10 domains retweeted by

	NB	CB	NH	CH
1	foxnews.com	nytimes.com	dailycaller.com	nytimes.com
2	dailycaller.com	cnn.com	foxnews.com	washingtonpost.com
3	breitbart.com	theguardian.com	breitbart.com	theguardian.com
4	dailywire.com	washingtonpost.com	justthenews.com	cnn.com
5	nypost.com	yahoo.com	dailywire.com	rawstory.com
6	justthenews.com	thehill.com	thegatewaypundit.com	thehill.com
7	thegatewaypundit.com	reuters.com	hannity.com	vox.com
8	CoronaVirus.gov	vox.com	politicususa.com	politico.com
9	politicususa.com	politico.com	nypost.com	independent.co.uk
10	hannity.com	cbsnews.com	CoronaVirus.gov	yahoo.com

(b) Top 10 users retweeted by

	NB	CB	NH	CH
1	@DailyCaller	@washingtonpost	@DailyCaller	@washingtonpost
2	@DonaldJTrumpJr	@thehill	@DonaldJTrumpJr	@thehill
3	@TrumpWarRoom	@CBSNews	@jsolomonReports	@HillaryClinton
4	@yogagenie	@guardian	@seanhannity	@TeaPainUSA
5	@realDonaldTrump	@realDonaldTrump	@TrumpWarRoom	@guardian
6	@Rparkerscience	@HillaryClinton	@marklevinshow	@JoeBiden
7	@seanhannity	@Independent	@realDonaldTrump	@BillKristol
8	@jsolomonReports	@voxdotcom	@RealJamesWoods	@Independent
9	@Bamafanaticfan1	@CNNPolitics	@thehill	@CNNPolitics
10	@thehill	@Thomas1774Paine	@Thomas1774Paine	@realDonaldTrump

Table 3: 5G

(a) Top 10 domains retweeted by

	NB	CB	NH	CH
**1**	beforeitsnews.com	bbc.co.uk	worldtruth.tv	theguardian.com
**2**	dailypost.ng	theguardian.com	express.co.uk	bbc.co.uk
**3**	worldtruth.tv	bbc.com	infowars.com	bbc.com
**4**	zerohedge.com	reuters.com	beforeitsnews.com	theverge.com
**5**	dailyrecord.co.uk	theverge.com	humansarefree.com	businessinsider.com
**6**	today.ng	cnn.com	neonnettle.com	cnn.com
**7**	infowars.com	businessinsider.com	thelastamericanvagabond.com	reuters.com
**8**	banned.video	nytimes.com	dailycaller.com	ft.com
**9**	thetruthaboutcancer.com	newsweek.com	paulcraigroberts.org	vox.com
**10**	rt.com	vox.com	thesun.co.uk	nytimes.com

(b) Top 10 users retweeted by

	NB	CB	NH	CH
**1**	@shinethelight17	@Reuters	@WorldTruthTV	@guardian
**2**	@DailyPostNGR	@guidaautonoma	@BILDERBERG_GP	@rooshv
**3**	@Laurel700	@Exchange5g	@davidicke	@guardiannews
**4**	@davidicke	@rooshv	@shinethelight17	@verge
**5**	@freezerohedge	@HaroldSinnott	@TornadoNewsLink	@Omojuwa
**6**	@NigeriaNewsdesk	@verge	@boblister_poole	@Reuters
**7**	@BANNEDdotVIDEO	@ipfconline1	@DailyPostNGR	@davidicke
**8**	@WorldTruthTV	@Shirastweet	@BANNEDdotVIDEO	@Exchange5g
**9**	@owhy3	@nuskitconsultan	@davidkurten	@davidkurten
**10**		@guardian	@buttscornershop	@ruskin147

Table 4: Bill Gates

(a) Top 10 domains retweeted by

	NB	CB	NH	CH
**1**	thegatewaypundit.com	thehill.com	thegatewaypundit.com	thehill.com
**2**	dailycaller.com	nytimes.com	activistpost.com	nytimes.com
**3**	newspunch.com	vox.com	express.co.uk	washingtonpost.com
**4**	nypost.com	washingtonpost.com	dailycaller.com	businessinsider.com
**5**	bloomberg.com	businessinsider.com	newspunch.com	vox.com
**6**	express.co.uk	wsj.com	nypost.com	theverge.com
**7**	americanthinker.com	cnn.com	worldtruth.tv	wsj.com
**8**	activistpost.com	theverge.com	zerohedge.com	newspunch.com
**9**	presstv.com	bbc.co.uk	montanadailygazette.com	cnn.com
**10**	naturalblaze.com	reuters.com	blacklistednews.com	thedailybeast.com

(b) Top 10 users retweeted by

	NB	CB	NH	CH
**1**	@EyesOnQ	@cjtruth	@o_rips	@cjtruth
**2**	@Ian56789	@EyesOnQ	@gatewaypundit	@EyesOnQ
**3**	@zsixkillerk	@joshrogin	@EyesOnQ	@carmindabrendel
**4**	@gatewaypundit	@WSJ	@bbusa617	@joshrogin
**5**	@gaye_gallops	@sapinker	@Ian56789	@WSJ
**6**	@taxfreeok	@HollyWilhelm4	@KarluskaP	@HollyWilhelm4
**7**	@SM0799	@businessinsider	@DailyCaller	@sapinker
**8**	@bbusa617	@carmindabrendel	@davidicke	@ezraklein
**9**	@DailyCaller	@voxdotcom	@WorldTruthTV	@stanveuger
**10**	@davidicke	@gaye_gallops	@zsixkillerk	@thehill
